# A monoclinic polymorph of *N*-eth­oxy­carbonyl-*N*′-(3-phenyl-1*H*-1,2,4-triazol-5-yl)thio­urea

**DOI:** 10.1107/S1600536810026164

**Published:** 2010-07-10

**Authors:** Anton V. Dolzhenko, Geok Kheng Tan, Anna V. Dolzhenko, Lip Lin Koh, Wai Keung Chui

**Affiliations:** a Department of Pharmacy, Faculty of Science, National University of Singapore, 18 Science Drive 4, Singapore 117543, Singapore; b Department of Chemistry, Faculty of Science, National University of Singapore, 3 Science Drive 3, Singapore 117543, Singapore; c Perm State Pharmaceutical Academy, 2 Polevaya Street, Perm 614990, Russian Federation

## Abstract

The title compound, C_12_H_13_N_5_O_2_S {systematic name: ethyl *N*-[*N*-(3-phenyl-1*H*-1,2,4-triazol-5-yl)carbamothio­yl]carbamate}, is a monoclinic polymorph (space group *P*2_1_/*c*) which crystallizes with three similar independent mol­ecules in the asymmetric unit. The triazole ring makes dihedral angles of 6.6 (2), 8.4 (2) and 10.6 (2)° with the phenyl ring in the three independent molecules. The structure was previously reported [Dolzhenko *et al.* (2010*a* ▶). *Acta Cryst.*, E**46**, o425] as a triclinic polymorph crystallizing in space group *P* 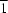. Mol­ecules in both polymorphs possess two *S*(6) rings generated by intra­molecular N—H⋯S and N—H⋯O hydrogen bonds, resulting in similar mol­ecular geometries. However, the two polymorphs differ in the crystal packing. In contrast to the dimers of the triclinic polymorph, mol­ecules of the monoclinic polymorph are connected by inter­molecular N—H⋯S and N—H⋯N hydrogen bonds, forming pseudosymmetric trimers arranged in sheets parallel to (302).

## Related literature

For the synthesis, tautomerism and crystal structure studies of related 1,2,4-triazoles, see: Dolzhenko *et al.* (2007 ▶, 2009**a* ▶,*b* ▶,c* ▶). For the crystal structure of the triclinic polymorph, see: Dolzhenko *et al.* (2010*a* ▶). For the crystal structure of *N*-carbeth­oxy-*N′*-(3-aryl-1*H*-1,2,4-triazol-5-yl)thio­urea, see: Dol­zhenko *et al.* (2010*b* ▶). For the graph-set analysis of hydrogen bonding, see: Bernstein *et al.* (1995 ▶).
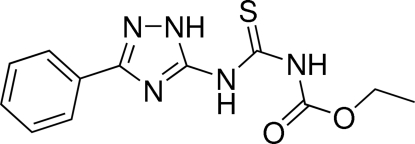


## Experimental

### 

#### Crystal data

C_12_H_13_N_5_O_2_S
*M* *_r_* = 291.33
Monoclinic, 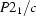

*a* = 13.4743 (6) Å
*b* = 20.4817 (9) Å
*c* = 15.0266 (7) Å
β = 104.040 (1)°
*V* = 4023.1 (3) Å^3^
*Z* = 12
Mo *K*α radiation
μ = 0.25 mm^−1^
*T* = 223 K
0.56 × 0.24 × 0.12 mm

#### Data collection

Bruker SMART APEX CCD diffractometer
Absorption correction: multi-scan (*SADABS*; Sheldrick, 2001 ▶) *T*_min_ = 0.872, *T*_max_ = 0.971
28189 measured reflections
9216 independent reflections
5772 reflections with *I* > 2σ(*I*)
*R*_int_ = 0.055

#### Refinement

*R*[*F* ^2^ > 2σ(*F* ^2^)] = 0.063
*wR*(*F* ^2^) = 0.164
*S* = 1.03
9216 reflections
598 parameters
86 restraints
H atoms treated by a mixture of independent and constrained refinement
Δρ_max_ = 0.85 e Å^−3^
Δρ_min_ = −0.52 e Å^−3^

### 

Data collection: *SMART* (Bruker, 2001 ▶); cell refinement: *SAINT* (Bruker, 2001 ▶); data reduction: *SAINT*; program(s) used to solve structure: *SHELXS97* (Sheldrick, 2008 ▶); program(s) used to refine structure: *SHELXS97* (Sheldrick, 2008 ▶); molecular graphics: *SHELXTL* (Sheldrick, 2008 ▶); software used to prepare material for publication: *SHELXTL*.

## Supplementary Material

Crystal structure: contains datablocks I, global. DOI: 10.1107/S1600536810026164/ci5124sup1.cif

Structure factors: contains datablocks I. DOI: 10.1107/S1600536810026164/ci5124Isup2.hkl

Additional supplementary materials:  crystallographic information; 3D view; checkCIF report

## Figures and Tables

**Table 1:** Hydrogen-bond geometry (Å, °)

*D*—H⋯*A*	*D*—H	H⋯*A*	*D*⋯*A*	*D*—H⋯*A*
N3—H3*N*⋯S1	0.90 (1)	2.44 (3)	2.978 (2)	119 (2)
N8—H8*N*⋯S2	0.90 (1)	2.42 (3)	2.975 (2)	121 (2)
N13—H13*N*⋯S3	0.90 (1)	2.44 (3)	2.997 (2)	120 (2)
N8—H8*N*⋯S3	0.90 (1)	2.44 (2)	3.253 (2)	150 (2)
N3—H3*N*⋯S2^i^	0.90 (2)	2.43 (2)	3.238 (2)	151 (2)
N13—H13*N*⋯S1^ii^	0.90 (2)	2.49 (3)	3.306 (3)	151 (2)
N4—H4*N*⋯O2	0.89 (1)	1.94 (2)	2.661 (3)	137 (2)
N9—H9*N*⋯O4	0.90 (1)	1.91 (2)	2.648 (3)	138 (2)
N14—H14*N*⋯O6	0.89 (1)	1.90 (2)	2.619 (3)	136 (2)
N10—H10*N*⋯N2^ii^	0.89 (2)	2.51 (2)	3.390 (3)	170 (2)
N15—H15*N*⋯N7	0.89 (2)	2.31 (2)	3.197 (3)	180 (3)

Symmetry codes: (i) 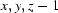; (ii) 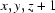.

**Fig. 1.**
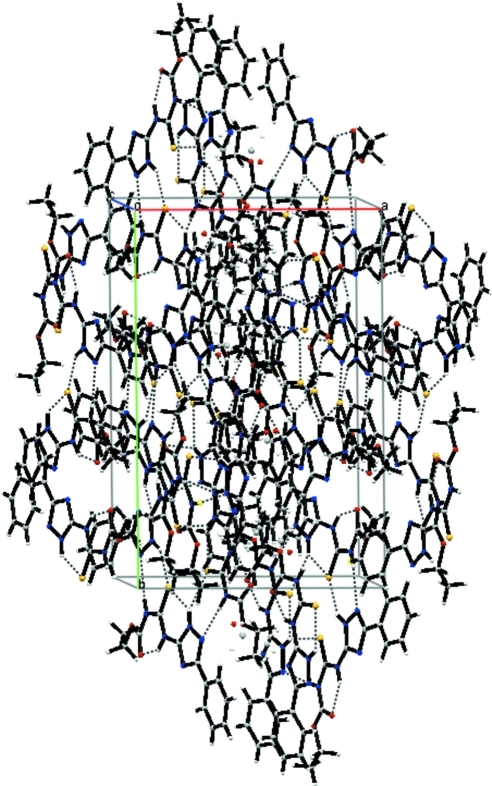
Crystal packing of the title compound, viewed along the c axis.

**Fig. 2.**
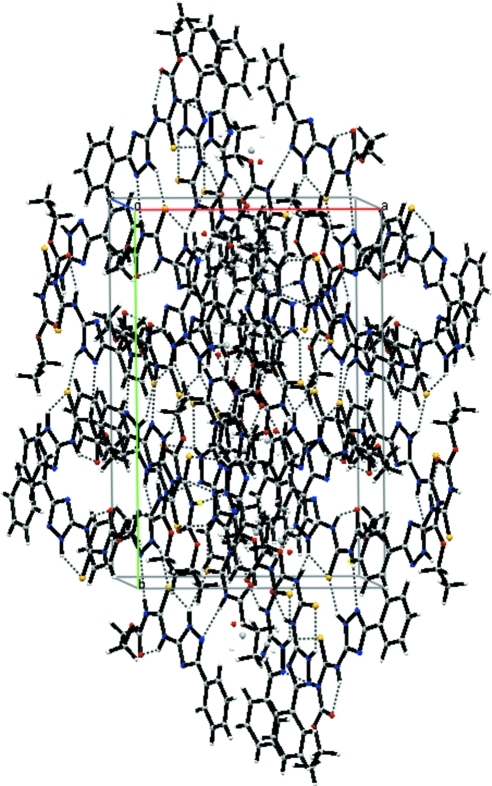
Synthesis of N-carbethoxy-N'-(3-phenyl-1H-1,2,4-triazol-5-yl)thiourea

## supplementary crystallographic information

### Comment

In our studies on the triazole tautomerism in solution and crystals (Dolzhenko *et al.*, 2009*a,b,c*), we reported recently the crystal structure of *N*-carbethoxy-*N'*-(3-phenyl-1*H*-1,2,4-triazol-5-yl)thiourea, which crystallized in the triclinic *P1*space group (Dolzhenko *et al.*, 2010*a*). Herein, we present a new monoclinic polymorph of *N*-carbethoxy-*N'*-(3-phenyl-1*H*-1,2,4-triazol-5-yl)thiourea, which crystallized in the *P2_1_/c* space group with three similar crystallographically independent molecules in the asymmetric unit (Fig. 1). The molecules in both polymorphs exist in the same tautomeric form and possess similar molecular geometry.

In both polymorph, the N—H···S hydrogen bonds between the endocyclic NH group of the triazole ring and the thioureido sulfur atom (Fig. 2 and Table 1) are arranged in a *S*6 graph-set motif (Bernstein *et al.*, 1995) stabilizing the tautomer structure. The general configurations of the carbethoxythiourea group in the title molecule replicate that of previously reported for triclinic polymorph (Dolzhenko *et al.*, 2010*a*) and a similar structure (Dolzhenko *et al.*, 2010*b*). The strong intramolecular hydrogen bonding between carbonyl oxygen atom and thiourea NH group arranged in *S*(6) graph-set motif which is common for carbethoxythioureas (Dolzhenko *et al.*, 2010*a,b*). In the three independent molecules, the triazole ring make dihedral angles of 6.6 (2)°, 8.4 (2)°  and 10.6 (2)° with the corresponding phenyl rings [*cf.* 6.59 (10)° for the triclinic polymorph (Dolzhenko *et al.*, 2010*a*)].

The monoclinic and triclinic polymorphs are significantly different in crystal packing. In contrast to the dimers of triclinic polymorph (Dolzhenko *et al.*, 2010*a*), molecules of monoclinic polymorph are connected with intermolecular hydrogen bonding of the C═S···H—N—N···H—N pattern forming pseudosymmetric trimer structures arranged in sheets parallel to the (302) plane.

### Experimental

The title compound was synthesized by nucleophilic addition of 3(5)-amino-5(3)-phenyl-1*H*-1,2,4-triazole (Dolzhenko *et al.*, 2007), to ethoxycarbonyl isothiocyanate in DMF solution at room temperature (Fig.3). Single crystals suitable for crystallographic analysis were grown by recrystallization from toluene.

### Refinement

All the H atoms attached to the carbon atoms were constrained in a riding motion approximation [0.94 Å for C_aryl_–H, 0.98 Å for methylene H atoms and 0.97 Å for methyl groups; *U*_iso_(H) = 1.2*U*_eq_(C_aryl_ and C_methylene_) and 1.5*U*_eq_(C_methyl_)] while the N-bound H atoms were located in a difference map and refined with restraints in bond length and thermal parameters. One of the ethoxy -OCH_2_CH_3_ group is disordered over two orientations with occupancies of 0.634 (7) and 0.366 (7). The corresponding bond distances in the disorder components were restrained to be the same. The U^ij^ parameters of disordered atoms were restrained to an approximate isotropic behaviour.

### Crystal data

**Table:**

C_12_H_13_N_5_O_2_S	*F*(000) = 1824
*M**_r_* = 291.33	*D*_x_ = 1.443 Mg m^−^^3^
Monoclinic, *P*2_1_/*c*	Melting point: 454 K
Hall symbol: -P 2ybc	Mo *K*α radiation, λ = 0.71073 Å
*a* = 13.4743 (6) Å	Cell parameters from 3587 reflections
*b* = 20.4817 (9) Å	θ = 2.4–21.7°
*c* = 15.0266 (7) Å	µ = 0.25 mm^−^^1^
β = 104.040 (1)°	*T* = 223 K
*V* = 4023.1 (3) Å^3^	Rod, colourless
*Z* = 12	0.56 × 0.24 × 0.12 mm

### Data collection

**Table:**

Bruker SMART APEX CCD diffractometer	9216 independent reflections
Radiation source: fine-focus sealed tube	5772 reflections with *I* > 2σ(*I*)
graphite	*R*_int_ = 0.055
φ and ω scans	θ_max_ = 27.5°, θ_min_ = 1.7°
Absorption correction: multi-scan (*SADABS*; Sheldrick, 2001)	*h* = −17→17
*T*_min_ = 0.872, *T*_max_ = 0.971	*k* = −25→26
28189 measured reflections	*l* = −11→19

### Refinement

**Table:**

Refinement on *F*^2^	Primary atom site location: structure-invariant direct methods
Least-squares matrix: full	Secondary atom site location: difference Fourier map
*R*[*F*^2^ > 2σ(*F*^2^)] = 0.063	Hydrogen site location: inferred from neighbouring sites
*wR*(*F*^2^) = 0.164	H atoms treated by a mixture of independent and constrained refinement
*S* = 1.03	*w* = 1/[σ^2^(*F*_o_^2^) + (0.0784*P*)^2^ + 0.4202*P*] where *P* = (*F*_o_^2^ + 2*F*_c_^2^)/3
9216 reflections	(Δ/σ)_max_ = 0.001
598 parameters	Δρ_max_ = 0.85 e Å^−^^3^
86 restraints	Δρ_min_ = −0.52 e Å^−^^3^

### Special details

**Table:**

Geometry. All e.s.d.'s (except the e.s.d. in the dihedral angle between two l.s. planes) are estimated using the full covariance matrix. The cell e.s.d.'s are taken into account individually in the estimation of e.s.d.'s in distances, angles and torsion angles; correlations between e.s.d.'s in cell parameters are only used when they are defined by crystal symmetry. An approximate (isotropic) treatment of cell e.s.d.'s is used for estimating e.s.d.'s involving l.s. planes.
Refinement. Refinement of *F*^2^ against ALL reflections. The weighted *R*-factor *wR* and goodness of fit *S* are based on *F*^2^, conventional *R*-factors *R* are based on *F*, with *F* set to zero for negative *F*^2^. The threshold expression of *F*^2^ > σ(*F*^2^) is used only for calculating *R*-factors(gt) *etc*. and is not relevant to the choice of reflections for refinement. *R*-factors based on *F*^2^ are statistically about twice as large as those based on *F*, and *R*- factors based on ALL data will be even larger.

### Fractional atomic coordinates and isotropic or equivalent isotropic displacement parameters (Å^2^)

**Table:**

	*x*	*y*	*z*	*U*_iso_*/*U*_eq_	Occ. (<1)
S1	0.20531 (6)	0.64994 (3)	−0.06103 (6)	0.0441 (2)	
S2	0.17227 (7)	0.48747 (3)	0.97238 (6)	0.0513 (2)	
S3	0.31718 (9)	0.53635 (4)	0.83353 (7)	0.0713 (3)	
O1	0.23376 (15)	0.88193 (8)	−0.06109 (14)	0.0410 (5)	
O2	0.17439 (15)	0.85011 (9)	0.06024 (14)	0.0404 (5)	
O3	0.04408 (16)	0.38003 (9)	1.20243 (14)	0.0455 (5)	
O4	0.06816 (16)	0.30621 (9)	1.09867 (14)	0.0445 (5)	
O6	0.4656 (2)	0.50555 (10)	0.59819 (18)	0.0682 (7)	
N1	0.07377 (17)	0.68052 (10)	0.18244 (15)	0.0328 (5)	
N2	0.07758 (18)	0.57196 (10)	0.15331 (16)	0.0369 (5)	
N3	0.11842 (18)	0.60701 (10)	0.09369 (16)	0.0347 (5)	
H3N	0.1465 (19)	0.5860 (12)	0.0535 (15)	0.042*	
N4	0.15156 (17)	0.72134 (10)	0.06799 (16)	0.0327 (5)	
H4N	0.144 (2)	0.7611 (7)	0.0893 (18)	0.039*	
N5	0.21787 (18)	0.77628 (10)	−0.03862 (16)	0.0354 (5)	
H5N	0.244 (2)	0.7715 (14)	−0.0874 (13)	0.043*	
N6	0.21916 (17)	0.28401 (10)	0.88026 (16)	0.0339 (5)	
N7	0.28571 (17)	0.35600 (10)	0.79634 (16)	0.0372 (5)	
N8	0.24310 (17)	0.38767 (10)	0.85782 (16)	0.0353 (5)	
H8N	0.243 (2)	0.4314 (5)	0.860 (2)	0.042*	
N9	0.15402 (17)	0.35768 (10)	0.97396 (16)	0.0340 (5)	
H9N	0.1309 (19)	0.3232 (9)	0.9998 (18)	0.041*	
N10	0.09302 (18)	0.41605 (10)	1.08206 (16)	0.0361 (5)	
H10N	0.087 (2)	0.4548 (8)	1.1074 (18)	0.043*	
N11	0.40967 (17)	0.69867 (10)	0.67246 (16)	0.0361 (5)	
N12	0.34161 (18)	0.74325 (10)	0.78232 (18)	0.0418 (6)	
N13	0.34239 (19)	0.67680 (10)	0.78753 (17)	0.0391 (6)	
H13N	0.318 (2)	0.6556 (13)	0.8300 (15)	0.047*	
N14	0.39497 (18)	0.58664 (10)	0.70279 (16)	0.0349 (5)	
H14N	0.421 (2)	0.5794 (14)	0.6543 (13)	0.042*	
N15	0.37852 (18)	0.47458 (10)	0.70418 (17)	0.0373 (6)	
H15N	0.353 (2)	0.4418 (10)	0.7300 (18)	0.045*	
C1	−0.0235 (2)	0.65393 (13)	0.3321 (2)	0.0389 (7)	
H1	−0.0021	0.6967	0.3239	0.047*	
C2	−0.0761 (2)	0.64147 (14)	0.3989 (2)	0.0455 (7)	
H2	−0.0906	0.6756	0.4355	0.055*	
C3	−0.1069 (2)	0.57897 (14)	0.4112 (2)	0.0483 (8)	
H3	−0.1420	0.5705	0.4569	0.058*	
C4	−0.0870 (2)	0.52842 (14)	0.3573 (2)	0.0462 (8)	
H4	−0.1090	0.4858	0.3659	0.055*	
C5	−0.0343 (2)	0.54099 (13)	0.2905 (2)	0.0409 (7)	
H5	−0.0203	0.5067	0.2538	0.049*	
C6	−0.0022 (2)	0.60396 (12)	0.27731 (19)	0.0328 (6)	
C7	0.0510 (2)	0.61851 (12)	0.20477 (19)	0.0316 (6)	
C8	0.11565 (19)	0.67033 (12)	0.11294 (18)	0.0303 (6)	
C9	0.1901 (2)	0.71755 (12)	−0.00656 (19)	0.0317 (6)	
C10	0.2060 (2)	0.83789 (12)	−0.0065 (2)	0.0336 (6)	
C11	0.2122 (2)	0.94956 (12)	−0.0410 (2)	0.0422 (7)	
H11A	0.2514	0.9618	0.0205	0.051*	
H11B	0.1392	0.9551	−0.0441	0.051*	
C12	0.2431 (2)	0.99114 (13)	−0.1123 (2)	0.0506 (8)	
H12A	0.3157	0.9858	−0.1076	0.061*	
H12B	0.2286	1.0366	−0.1024	0.061*	
H12C	0.2049	0.9778	−0.1729	0.061*	
C13	0.2936 (2)	0.17599 (13)	0.7891 (2)	0.0432 (7)	
H13	0.2675	0.1673	0.8404	0.052*	
C14	0.3236 (3)	0.12477 (14)	0.7417 (2)	0.0517 (8)	
H14	0.3181	0.0817	0.7614	0.062*	
C15	0.3612 (2)	0.13640 (14)	0.6660 (2)	0.0491 (8)	
H15	0.3809	0.1015	0.6334	0.059*	
C16	0.3697 (3)	0.20008 (15)	0.6385 (2)	0.0531 (8)	
H16	0.3960	0.2086	0.5872	0.064*	
C17	0.3401 (2)	0.25111 (14)	0.6854 (2)	0.0460 (7)	
H17	0.3459	0.2942	0.6656	0.055*	
C18	0.3016 (2)	0.23983 (12)	0.7617 (2)	0.0348 (6)	
C19	0.2690 (2)	0.29402 (12)	0.81255 (19)	0.0333 (6)	
C20	0.2040 (2)	0.34429 (12)	0.90587 (19)	0.0317 (6)	
C21	0.1390 (2)	0.41679 (12)	1.00938 (19)	0.0329 (6)	
C22	0.0671 (2)	0.36185 (13)	1.1259 (2)	0.0366 (6)	
C23	0.0197 (3)	0.32624 (14)	1.2587 (2)	0.0491 (8)	
H23A	0.0681	0.2902	1.2611	0.059*	
H23B	−0.0495	0.3099	1.2323	0.059*	
C24	0.0270 (3)	0.35195 (17)	1.3519 (3)	0.0707 (11)	
H24A	0.0939	0.3714	1.3754	0.085*	
H24B	0.0174	0.3166	1.3919	0.085*	
H24C	−0.0255	0.3847	1.3496	0.085*	
C25	0.4554 (2)	0.82809 (14)	0.6144 (2)	0.0499 (8)	
H25	0.4852	0.7915	0.5933	0.060*	
C26	0.4684 (3)	0.88965 (16)	0.5801 (3)	0.0598 (9)	
H26	0.5072	0.8948	0.5364	0.072*	
C27	0.4241 (3)	0.94289 (15)	0.6103 (3)	0.0633 (10)	
H27	0.4321	0.9846	0.5869	0.076*	
C28	0.3683 (3)	0.93515 (15)	0.6747 (3)	0.0591 (10)	
H28	0.3381	0.9718	0.6951	0.071*	
C29	0.3559 (2)	0.87429 (13)	0.7100 (2)	0.0485 (8)	
H29	0.3181	0.8697	0.7546	0.058*	
C30	0.3992 (2)	0.82008 (13)	0.6793 (2)	0.0403 (7)	
C31	0.3835 (2)	0.75410 (12)	0.7126 (2)	0.0357 (6)	
C32	0.3826 (2)	0.65233 (12)	0.72182 (19)	0.0327 (6)	
C33	0.3652 (2)	0.53406 (12)	0.7427 (2)	0.0353 (6)	
C34	0.4265 (3)	0.46419 (14)	0.6349 (2)	0.0472 (8)	
O5	0.4364 (4)	0.4001 (3)	0.6227 (3)	0.0402 (13)	0.634 (7)
C35	0.4867 (5)	0.3865 (3)	0.5460 (5)	0.0637 (17)	0.634 (7)
H35A	0.4571	0.4146	0.4935	0.076*	0.634 (7)
H35B	0.5600	0.3959	0.5660	0.076*	0.634 (7)
C36	0.4716 (6)	0.3202 (3)	0.5201 (5)	0.0745 (18)	0.634 (7)
H36A	0.5042	0.3111	0.4705	0.089*	0.634 (7)
H36B	0.3989	0.3112	0.4998	0.089*	0.634 (7)
H36C	0.5015	0.2926	0.5722	0.089*	0.634 (7)
O5A	0.3960 (7)	0.4021 (5)	0.5995 (6)	0.041 (2)	0.366 (7)
C35A	0.4221 (8)	0.3748 (5)	0.5141 (6)	0.076 (3)	0.366 (7)
H35C	0.3752	0.3392	0.4884	0.091*	0.366 (7)
H35D	0.4166	0.4089	0.4675	0.091*	0.366 (7)
C36A	0.5241 (7)	0.3513 (7)	0.5415 (9)	0.088 (3)	0.366 (7)
H36D	0.5445	0.3340	0.4886	0.106*	0.366 (7)
H36E	0.5281	0.3171	0.5869	0.106*	0.366 (7)
H36F	0.5694	0.3868	0.5679	0.106*	0.366 (7)

### Atomic displacement parameters (Å^2^)

**Table:**

	*U*^11^	*U*^22^	*U*^33^	*U*^12^	*U*^13^	*U*^23^
S1	0.0679 (5)	0.0275 (3)	0.0481 (5)	0.0027 (3)	0.0359 (4)	−0.0004 (3)
S2	0.0890 (6)	0.0220 (3)	0.0586 (5)	−0.0087 (3)	0.0485 (5)	−0.0055 (3)
S3	0.1328 (9)	0.0264 (4)	0.0852 (7)	−0.0089 (4)	0.0859 (7)	−0.0078 (4)
O1	0.0559 (12)	0.0216 (9)	0.0544 (13)	−0.0001 (8)	0.0309 (10)	0.0031 (9)
O2	0.0540 (12)	0.0286 (9)	0.0454 (12)	−0.0025 (8)	0.0254 (10)	−0.0023 (9)
O3	0.0715 (14)	0.0304 (10)	0.0441 (12)	−0.0072 (9)	0.0322 (11)	0.0013 (9)
O4	0.0625 (13)	0.0262 (10)	0.0512 (13)	−0.0044 (9)	0.0263 (11)	0.0021 (9)
O6	0.121 (2)	0.0318 (11)	0.0786 (17)	0.0009 (12)	0.0760 (16)	0.0008 (12)
N1	0.0418 (13)	0.0264 (11)	0.0341 (13)	0.0006 (9)	0.0166 (10)	−0.0002 (10)
N2	0.0496 (14)	0.0283 (11)	0.0377 (13)	−0.0019 (10)	0.0198 (11)	0.0033 (10)
N3	0.0477 (14)	0.0252 (11)	0.0378 (13)	0.0000 (10)	0.0229 (11)	−0.0001 (10)
N4	0.0452 (13)	0.0216 (10)	0.0368 (13)	0.0009 (9)	0.0207 (11)	0.0000 (10)
N5	0.0494 (14)	0.0254 (11)	0.0393 (14)	0.0012 (10)	0.0261 (11)	0.0027 (10)
N6	0.0449 (13)	0.0208 (10)	0.0377 (13)	0.0008 (9)	0.0135 (11)	−0.0021 (10)
N7	0.0485 (14)	0.0272 (11)	0.0408 (14)	−0.0003 (10)	0.0204 (11)	−0.0054 (11)
N8	0.0501 (14)	0.0213 (10)	0.0411 (14)	−0.0016 (10)	0.0235 (11)	−0.0023 (10)
N9	0.0490 (14)	0.0192 (10)	0.0383 (13)	−0.0029 (9)	0.0198 (11)	0.0004 (10)
N10	0.0510 (14)	0.0240 (11)	0.0402 (14)	0.0000 (10)	0.0242 (11)	−0.0002 (10)
N11	0.0446 (14)	0.0246 (11)	0.0413 (14)	−0.0028 (9)	0.0146 (11)	0.0024 (10)
N12	0.0541 (15)	0.0229 (11)	0.0496 (16)	−0.0003 (10)	0.0148 (13)	−0.0021 (11)
N13	0.0577 (16)	0.0243 (11)	0.0412 (15)	−0.0009 (10)	0.0235 (12)	−0.0014 (11)
N14	0.0503 (14)	0.0232 (10)	0.0381 (14)	−0.0002 (10)	0.0244 (11)	−0.0007 (10)
N15	0.0537 (15)	0.0208 (11)	0.0461 (15)	−0.0024 (10)	0.0287 (12)	−0.0028 (10)
C1	0.0518 (18)	0.0317 (14)	0.0360 (16)	−0.0018 (12)	0.0163 (13)	0.0029 (13)
C2	0.066 (2)	0.0379 (16)	0.0387 (17)	0.0036 (14)	0.0235 (15)	0.0006 (14)
C3	0.071 (2)	0.0423 (17)	0.0416 (17)	0.0021 (15)	0.0328 (16)	0.0105 (15)
C4	0.063 (2)	0.0311 (14)	0.0522 (19)	−0.0042 (13)	0.0298 (16)	0.0100 (14)
C5	0.0547 (18)	0.0312 (14)	0.0425 (17)	−0.0002 (12)	0.0232 (14)	0.0013 (13)
C6	0.0382 (15)	0.0285 (13)	0.0336 (15)	−0.0001 (11)	0.0127 (12)	0.0050 (12)
C7	0.0368 (15)	0.0268 (13)	0.0335 (15)	0.0009 (11)	0.0129 (12)	0.0015 (12)
C8	0.0344 (14)	0.0258 (12)	0.0327 (15)	0.0015 (10)	0.0118 (12)	0.0001 (12)
C9	0.0354 (14)	0.0254 (12)	0.0378 (15)	−0.0005 (10)	0.0155 (12)	0.0045 (12)
C10	0.0333 (15)	0.0265 (13)	0.0437 (17)	−0.0017 (10)	0.0146 (13)	0.0004 (12)
C11	0.0544 (18)	0.0239 (13)	0.0523 (19)	0.0022 (12)	0.0210 (15)	0.0014 (13)
C12	0.062 (2)	0.0268 (14)	0.071 (2)	0.0003 (13)	0.0310 (18)	0.0062 (15)
C13	0.0514 (18)	0.0285 (14)	0.0544 (19)	0.0027 (12)	0.0219 (15)	−0.0029 (14)
C14	0.068 (2)	0.0262 (14)	0.066 (2)	0.0040 (14)	0.0261 (18)	−0.0050 (15)
C15	0.056 (2)	0.0363 (16)	0.056 (2)	0.0050 (14)	0.0173 (16)	−0.0157 (15)
C16	0.069 (2)	0.0500 (19)	0.048 (2)	−0.0010 (16)	0.0298 (17)	−0.0092 (16)
C17	0.065 (2)	0.0281 (14)	0.0504 (19)	−0.0030 (13)	0.0243 (16)	−0.0037 (14)
C18	0.0348 (15)	0.0277 (13)	0.0422 (17)	−0.0012 (11)	0.0099 (12)	−0.0042 (12)
C19	0.0367 (15)	0.0278 (13)	0.0353 (16)	−0.0022 (11)	0.0085 (12)	−0.0011 (12)
C20	0.0347 (15)	0.0257 (12)	0.0340 (15)	−0.0011 (10)	0.0068 (12)	−0.0009 (12)
C21	0.0406 (15)	0.0252 (12)	0.0350 (15)	0.0001 (11)	0.0133 (12)	−0.0004 (12)
C22	0.0437 (16)	0.0321 (15)	0.0377 (16)	−0.0013 (12)	0.0169 (13)	0.0031 (13)
C23	0.066 (2)	0.0380 (16)	0.0505 (19)	−0.0121 (14)	0.0289 (17)	0.0066 (15)
C24	0.116 (3)	0.052 (2)	0.062 (2)	−0.011 (2)	0.059 (2)	0.0019 (19)
C25	0.0508 (19)	0.0303 (15)	0.069 (2)	−0.0012 (13)	0.0158 (17)	0.0057 (15)
C26	0.062 (2)	0.0415 (18)	0.076 (3)	−0.0066 (15)	0.0177 (19)	0.0157 (18)
C27	0.070 (2)	0.0277 (16)	0.084 (3)	−0.0066 (15)	0.004 (2)	0.0120 (18)
C28	0.073 (2)	0.0249 (15)	0.073 (3)	0.0055 (15)	0.005 (2)	−0.0047 (16)
C29	0.057 (2)	0.0295 (15)	0.056 (2)	0.0001 (13)	0.0076 (16)	−0.0052 (14)
C30	0.0403 (16)	0.0268 (13)	0.0480 (18)	−0.0036 (12)	−0.0004 (14)	0.0009 (13)
C31	0.0388 (16)	0.0237 (13)	0.0425 (17)	−0.0014 (11)	0.0055 (13)	−0.0012 (12)
C32	0.0366 (15)	0.0248 (12)	0.0374 (15)	−0.0012 (11)	0.0101 (12)	−0.0020 (12)
C33	0.0439 (16)	0.0266 (13)	0.0418 (16)	−0.0023 (11)	0.0227 (13)	−0.0036 (12)
C34	0.069 (2)	0.0275 (14)	0.054 (2)	0.0017 (14)	0.0334 (17)	−0.0047 (14)
O5	0.047 (3)	0.0255 (19)	0.053 (3)	0.002 (2)	0.021 (2)	−0.010 (2)
C35	0.067 (4)	0.050 (3)	0.092 (4)	−0.011 (3)	0.056 (3)	−0.030 (3)
C36	0.089 (4)	0.064 (3)	0.086 (4)	−0.012 (3)	0.051 (3)	−0.016 (3)
O5A	0.060 (6)	0.032 (3)	0.026 (3)	0.022 (4)	0.001 (4)	−0.001 (3)
C35A	0.077 (5)	0.058 (4)	0.096 (5)	−0.006 (4)	0.028 (4)	0.024 (4)
C36A	0.083 (5)	0.080 (6)	0.107 (5)	−0.016 (5)	0.034 (5)	0.001 (5)

### Geometric parameters (Å, °)

**Table:**

S1—C9	1.647 (3)	C5—C6	1.390 (4)
S2—C21	1.651 (3)	C5—H5	0.94
S3—C33	1.647 (3)	C6—C7	1.473 (4)
O1—C10	1.332 (3)	C11—C12	1.503 (4)
O1—C11	1.462 (3)	C11—H11A	0.98
O2—C10	1.208 (3)	C11—H11B	0.98
O3—C22	1.316 (3)	C12—H12A	0.97
O3—C23	1.474 (3)	C12—H12B	0.97
O4—C22	1.212 (3)	C12—H12C	0.97
O6—C34	1.200 (3)	C13—C14	1.382 (4)
N1—C8	1.318 (3)	C13—C18	1.383 (4)
N1—C7	1.367 (3)	C13—H13	0.94
N2—C7	1.330 (3)	C14—C15	1.374 (4)
N2—N3	1.364 (3)	C14—H14	0.94
N3—C8	1.331 (3)	C15—C16	1.381 (4)
N3—H3N	0.897 (10)	C15—H15	0.94
N4—C9	1.346 (3)	C16—C17	1.373 (4)
N4—C8	1.393 (3)	C16—H16	0.94
N4—H4N	0.889 (10)	C17—C18	1.387 (4)
N5—C10	1.374 (3)	C17—H17	0.94
N5—C9	1.381 (3)	C18—C19	1.473 (4)
N5—H5N	0.890 (10)	C23—C24	1.476 (5)
N6—C20	1.324 (3)	C23—H23A	0.98
N6—C19	1.364 (3)	C23—H23B	0.98
N7—C19	1.322 (3)	C24—H24A	0.97
N7—N8	1.364 (3)	C24—H24B	0.97
N8—C20	1.331 (3)	C24—H24C	0.97
N8—H8N	0.897 (10)	C25—C30	1.382 (4)
N9—C21	1.357 (3)	C25—C26	1.389 (4)
N9—C20	1.382 (3)	C25—H25	0.94
N9—H9N	0.896 (10)	C26—C27	1.372 (5)
N10—C22	1.378 (3)	C26—H26	0.94
N10—C21	1.380 (3)	C27—C28	1.371 (5)
N10—H10N	0.892 (10)	C27—H27	0.94
N11—C32	1.310 (3)	C28—C29	1.381 (4)
N11—C31	1.371 (3)	C28—H28	0.94
N12—C31	1.324 (4)	C29—C30	1.384 (4)
N12—N13	1.363 (3)	C29—H29	0.94
N13—C32	1.334 (3)	C30—C31	1.474 (4)
N13—H13N	0.896 (10)	C34—O5	1.337 (6)
N14—C33	1.341 (3)	C34—O5A	1.401 (12)
N14—C32	1.394 (3)	O5—C35	1.499 (6)
N14—H14N	0.891 (10)	C35—C36	1.414 (6)
N15—C34	1.369 (4)	C35—H35A	0.98
N15—C33	1.379 (3)	C35—H35B	0.98
N15—H15N	0.883 (10)	C36—H36A	0.97
C1—C2	1.384 (4)	C36—H36B	0.97
C1—C6	1.387 (4)	C36—H36C	0.97
C1—H1	0.94	O5A—C35A	1.517 (7)
C2—C3	1.372 (4)	C35A—C36A	1.420 (7)
C2—H2	0.94	C35A—H35C	0.98
C3—C4	1.380 (4)	C35A—H35D	0.98
C3—H3	0.94	C36A—H36D	0.97
C4—C5	1.388 (4)	C36A—H36E	0.97
C4—H4	0.94	C36A—H36F	0.97
			
C10—O1—C11	114.5 (2)	C16—C17—C18	120.7 (3)
C22—O3—C23	115.0 (2)	C16—C17—H17	119.6
C8—N1—C7	102.2 (2)	C18—C17—H17	119.6
C7—N2—N3	102.3 (2)	C13—C18—C17	118.4 (3)
C8—N3—N2	109.5 (2)	C13—C18—C19	120.2 (3)
C8—N3—H3N	130.7 (18)	C17—C18—C19	121.4 (2)
N2—N3—H3N	119.6 (18)	N7—C19—N6	114.7 (2)
C9—N4—C8	127.6 (2)	N7—C19—C18	122.9 (2)
C9—N4—H4N	116.9 (19)	N6—C19—C18	122.4 (2)
C8—N4—H4N	115.4 (19)	N6—C20—N8	110.8 (2)
C10—N5—C9	127.8 (2)	N6—C20—N9	122.5 (2)
C10—N5—H5N	119.5 (19)	N8—C20—N9	126.7 (2)
C9—N5—H5N	112.6 (19)	N9—C21—N10	116.0 (2)
C20—N6—C19	102.4 (2)	N9—C21—S2	124.9 (2)
C19—N7—N8	102.3 (2)	N10—C21—S2	119.12 (19)
C20—N8—N7	109.7 (2)	O4—C22—O3	125.9 (3)
C20—N8—H8N	130 (2)	O4—C22—N10	124.8 (3)
N7—N8—H8N	121 (2)	O3—C22—N10	109.3 (2)
C21—N9—C20	127.8 (2)	O3—C23—C24	107.8 (2)
C21—N9—H9N	115.6 (18)	O3—C23—H23A	110.2
C20—N9—H9N	116.5 (18)	C24—C23—H23A	110.2
C22—N10—C21	127.0 (2)	O3—C23—H23B	110.2
C22—N10—H10N	116.9 (19)	C24—C23—H23B	110.2
C21—N10—H10N	115.7 (19)	H23A—C23—H23B	108.5
C32—N11—C31	102.4 (2)	C23—C24—H24A	109.5
C31—N12—N13	102.4 (2)	C23—C24—H24B	109.5
C32—N13—N12	109.4 (2)	H24A—C24—H24B	109.5
C32—N13—H13N	128.9 (19)	C23—C24—H24C	109.5
N12—N13—H13N	121.8 (19)	H24A—C24—H24C	109.5
C33—N14—C32	128.3 (2)	H24B—C24—H24C	109.5
C33—N14—H14N	116.7 (19)	C30—C25—C26	120.7 (3)
C32—N14—H14N	114.7 (19)	C30—C25—H25	119.7
C34—N15—C33	126.2 (2)	C26—C25—H25	119.7
C34—N15—H15N	121.2 (19)	C27—C26—C25	119.6 (4)
C33—N15—H15N	112.7 (19)	C27—C26—H26	120.2
C2—C1—C6	120.7 (3)	C25—C26—H26	120.2
C2—C1—H1	119.7	C28—C27—C26	119.9 (3)
C6—C1—H1	119.7	C28—C27—H27	120.0
C3—C2—C1	119.7 (3)	C26—C27—H27	120.0
C3—C2—H2	120.2	C27—C28—C29	120.9 (3)
C1—C2—H2	120.2	C27—C28—H28	119.5
C2—C3—C4	120.8 (3)	C29—C28—H28	119.5
C2—C3—H3	119.6	C28—C29—C30	119.7 (3)
C4—C3—H3	119.6	C28—C29—H29	120.1
C3—C4—C5	119.5 (3)	C30—C29—H29	120.1
C3—C4—H4	120.3	C25—C30—C29	119.1 (3)
C5—C4—H4	120.3	C25—C30—C31	119.8 (3)
C4—C5—C6	120.5 (3)	C29—C30—C31	121.0 (3)
C4—C5—H5	119.8	N12—C31—N11	114.4 (2)
C6—C5—H5	119.8	N12—C31—C30	123.1 (3)
C1—C6—C5	118.9 (3)	N11—C31—C30	122.5 (3)
C1—C6—C7	119.9 (2)	N11—C32—N13	111.5 (2)
C5—C6—C7	121.2 (2)	N11—C32—N14	121.3 (2)
N2—C7—N1	114.5 (2)	N13—C32—N14	127.2 (2)
N2—C7—C6	122.2 (2)	N14—C33—N15	116.0 (2)
N1—C7—C6	123.2 (2)	N14—C33—S3	124.6 (2)
N1—C8—N3	111.5 (2)	N15—C33—S3	119.4 (2)
N1—C8—N4	122.1 (2)	O6—C34—O5	124.1 (3)
N3—C8—N4	126.4 (2)	O6—C34—N15	125.5 (3)
N4—C9—N5	115.6 (2)	O5—C34—N15	109.7 (3)
N4—C9—S1	125.57 (19)	O6—C34—O5A	125.9 (5)
N5—C9—S1	118.8 (2)	N15—C34—O5A	106.3 (4)
O2—C10—O1	125.4 (2)	C34—O5—C35	111.5 (5)
O2—C10—N5	125.2 (2)	C36—C35—O5	109.2 (5)
O1—C10—N5	109.4 (2)	C36—C35—H35A	109.8
O1—C11—C12	106.7 (2)	O5—C35—H35A	109.8
O1—C11—H11A	110.4	C36—C35—H35B	109.8
C12—C11—H11A	110.4	O5—C35—H35B	109.8
O1—C11—H11B	110.4	H35A—C35—H35B	108.3
C12—C11—H11B	110.4	C35—C36—H36A	109.5
H11A—C11—H11B	108.6	C35—C36—H36B	109.5
C11—C12—H12A	109.5	H36A—C36—H36B	109.5
C11—C12—H12B	109.5	C35—C36—H36C	109.5
H12A—C12—H12B	109.5	H36A—C36—H36C	109.5
C11—C12—H12C	109.5	H36B—C36—H36C	109.5
H12A—C12—H12C	109.5	C34—O5A—C35A	123.3 (9)
H12B—C12—H12C	109.5	C36A—C35A—O5A	106.6 (6)
C14—C13—C18	120.7 (3)	C36A—C35A—H35C	110.4
C14—C13—H13	119.6	O5A—C35A—H35C	110.4
C18—C13—H13	119.6	C36A—C35A—H35D	110.4
C15—C14—C13	120.5 (3)	O5A—C35A—H35D	110.4
C15—C14—H14	119.7	H35C—C35A—H35D	108.6
C13—C14—H14	119.7	C35A—C36A—H36D	109.5
C14—C15—C16	119.0 (3)	C35A—C36A—H36E	109.5
C14—C15—H15	120.5	H36D—C36A—H36E	109.5
C16—C15—H15	120.5	C35A—C36A—H36F	109.5
C17—C16—C15	120.7 (3)	H36D—C36A—H36F	109.5
C17—C16—H16	119.7	H36E—C36A—H36F	109.5
C15—C16—H16	119.7		
			
C7—N2—N3—C8	0.9 (3)	C19—N6—C20—N9	179.4 (2)
C19—N7—N8—C20	−0.2 (3)	N7—N8—C20—N6	0.6 (3)
C31—N12—N13—C32	−0.6 (3)	N7—N8—C20—N9	−179.5 (2)
C6—C1—C2—C3	0.3 (5)	C21—N9—C20—N6	176.1 (3)
C1—C2—C3—C4	−0.6 (5)	C21—N9—C20—N8	−3.8 (5)
C2—C3—C4—C5	0.6 (5)	C20—N9—C21—N10	−175.3 (3)
C3—C4—C5—C6	−0.3 (5)	C20—N9—C21—S2	4.2 (4)
C2—C1—C6—C5	0.0 (4)	C22—N10—C21—N9	4.6 (4)
C2—C1—C6—C7	178.1 (3)	C22—N10—C21—S2	−175.0 (2)
C4—C5—C6—C1	0.0 (4)	C23—O3—C22—O4	1.2 (4)
C4—C5—C6—C7	−178.1 (3)	C23—O3—C22—N10	−177.1 (2)
N3—N2—C7—N1	−0.9 (3)	C21—N10—C22—O4	−10.9 (5)
N3—N2—C7—C6	176.8 (2)	C21—N10—C22—O3	167.5 (3)
C8—N1—C7—N2	0.6 (3)	C22—O3—C23—C24	163.0 (3)
C8—N1—C7—C6	−177.2 (2)	C30—C25—C26—C27	−0.6 (5)
C1—C6—C7—N2	176.7 (3)	C25—C26—C27—C28	0.6 (5)
C5—C6—C7—N2	−5.2 (4)	C26—C27—C28—C29	0.1 (5)
C1—C6—C7—N1	−5.7 (4)	C27—C28—C29—C30	−0.8 (5)
C5—C6—C7—N1	172.4 (3)	C26—C25—C30—C29	0.0 (5)
C7—N1—C8—N3	0.1 (3)	C26—C25—C30—C31	177.9 (3)
C7—N1—C8—N4	−179.7 (2)	C28—C29—C30—C25	0.7 (5)
N2—N3—C8—N1	−0.7 (3)	C28—C29—C30—C31	−177.2 (3)
N2—N3—C8—N4	179.1 (2)	N13—N12—C31—N11	0.7 (3)
C9—N4—C8—N1	−175.8 (3)	N13—N12—C31—C30	179.7 (2)
C9—N4—C8—N3	4.4 (4)	C32—N11—C31—N12	−0.6 (3)
C8—N4—C9—N5	178.4 (2)	C32—N11—C31—C30	−179.6 (2)
C8—N4—C9—S1	−1.1 (4)	C25—C30—C31—N12	171.4 (3)
C10—N5—C9—N4	−3.7 (4)	C29—C30—C31—N12	−10.6 (4)
C10—N5—C9—S1	175.9 (2)	C25—C30—C31—N11	−9.6 (4)
C11—O1—C10—O2	−6.9 (4)	C29—C30—C31—N11	168.3 (3)
C11—O1—C10—N5	172.5 (2)	C31—N11—C32—N13	0.1 (3)
C9—N5—C10—O2	5.1 (5)	C31—N11—C32—N14	179.1 (2)
C9—N5—C10—O1	−174.3 (2)	N12—N13—C32—N11	0.3 (3)
C10—O1—C11—C12	−176.9 (2)	N12—N13—C32—N14	−178.6 (3)
C18—C13—C14—C15	−0.5 (5)	C33—N14—C32—N11	−175.2 (3)
C13—C14—C15—C16	0.7 (5)	C33—N14—C32—N13	3.6 (5)
C14—C15—C16—C17	−0.6 (5)	C32—N14—C33—N15	173.9 (3)
C15—C16—C17—C18	0.4 (5)	C32—N14—C33—S3	−7.3 (4)
C14—C13—C18—C17	0.3 (4)	C34—N15—C33—N14	5.8 (4)
C14—C13—C18—C19	179.8 (3)	C34—N15—C33—S3	−173.0 (3)
C16—C17—C18—C13	−0.2 (5)	C33—N15—C34—O6	1.1 (6)
C16—C17—C18—C19	−179.7 (3)	C33—N15—C34—O5	172.4 (3)
N8—N7—C19—N6	−0.2 (3)	C33—N15—C34—O5A	−162.6 (4)
N8—N7—C19—C18	179.8 (2)	O6—C34—O5—C35	−9.8 (7)
C20—N6—C19—N7	0.6 (3)	N15—C34—O5—C35	178.8 (4)
C20—N6—C19—C18	−179.4 (2)	O5A—C34—O5—C35	93.0 (14)
C13—C18—C19—N7	172.0 (3)	C34—O5—C35—C36	−164.9 (7)
C17—C18—C19—N7	−8.6 (4)	O6—C34—O5A—C35A	8.9 (10)
C13—C18—C19—N6	−8.0 (4)	O5—C34—O5A—C35A	−85.4 (14)
C17—C18—C19—N6	171.4 (3)	N15—C34—O5A—C35A	172.6 (6)
C19—N6—C20—N8	−0.7 (3)	C34—O5A—C35A—C36A	80.4 (13)

### Hydrogen-bond geometry (Å, °)

**Table:**

*D*—H···*A*	*D*—H	H···*A*	*D*···*A*	*D*—H···*A*
N3—H3N···S1	0.90 (1)	2.44 (3)	2.978 (2)	119 (2)
N8—H8N···S2	0.90 (1)	2.42 (3)	2.975 (2)	121 (2)
N13—H13N···S3	0.90 (1)	2.44 (3)	2.997 (2)	120 (2)
N8—H8N···S3	0.90 (1)	2.44 (2)	3.253 (2)	150 (2)
N3—H3N···S2^i^	0.90 (2)	2.43 (2)	3.238 (2)	151 (2)
N13—H13N···S1^ii^	0.90 (2)	2.49 (3)	3.306 (3)	151 (2)
N4—H4N···O2	0.89 (1)	1.94 (2)	2.661 (3)	137 (2)
N9—H9N···O4	0.90 (1)	1.91 (2)	2.648 (3)	138 (2)
N14—H14N···O6	0.89 (1)	1.90 (2)	2.619 (3)	136 (2)
N10—H10N···N2^ii^	0.89 (2)	2.51 (2)	3.390 (3)	170 (2)
N15—H15N···N7	0.89 (2)	2.31 (2)	3.197 (3)	180 (3)

Symmetry codes: (i) *x*, *y*, *z*−1; (ii) *x*, *y*, *z*+1.
